# N1‐methylpseudouridine modification level correlates with protein expression, immunogenicity, and stability of mRNA

**DOI:** 10.1002/mco2.691

**Published:** 2024-09-17

**Authors:** Shaoyi Chen, Zheng Liu, Jingsheng Cai, Haoran Li, Mantang Qiu

**Affiliations:** ^1^ Department of Thoracic Surgery Thoracic Oncology Institute and Research Unit of Intelligence Diagnosis and Treatment in Early Non‐small Cell Lung Cancer Peking University People's Hospital Beijing China; ^2^ Institute of Advanced Clinical Medicine Peking University Beijing China

Dear Editor,

Messenger RNA (mRNA) has undergone significant evolution, emerging as a robust platform for diverse therapeutic applications such as vaccines, protein replacement, and adoptive cell therapy across infectious, cancer, and immunological diseases. In vitro transcribed mRNA offers distinct advantages, including cytoplasmic transient expression, devoid of the risk of genomic integration. Nucleotide modifications, such as pseudouridine (Ψ), N1‐methylpseudouridine (m1Ψ), and 5‐methylcytidine (m5C), play pivotal roles in mRNA immunogenicity, stability, and translational efficiency. Notably, standing out as a state‐of‐the‐art modification, global m1Ψ modification has been performed in the two approved mRNA vaccines against severe acute respiratory syndrome coronavirus 2, mRNA‐1273, and BNT162b2. The in vivo stability and translational duration of synthesized mRNA are partially restricted by its immunogenicity. Recognition of foreign RNA by toll‐like receptor 3 (TLR3), TLR7, TLR8, and retinoic acid‐inducible gene I (RIG‐I)‐like receptors triggers innate immunity, leading to RNA degradation.^1^, ^2^ The therapeutic application of mRNA necessitates a delicate balance between immune activation and protein expression. For infectious disease vaccines, chemical modifications can minimize inflammatory responses while ensuring effective mRNA translation. Conversely, cancer vaccines require adequate innate immune stimulation for anti‐tumor immunity. Thus far, most studies have investigated alternative m1Ψ modification individually or combined with others. However, it is unknown whether m1Ψ modification ratio in mRNA has an impact on the delicate balance between immunogenicity, stability, and translational efficiency.

Therefore, we synthesized mRNA encoding enhanced green fluorescent protein (mEGFP) with different m1Ψ modification ratios (5%, 10%, 20%, 50%, 75%, and 100%) through in vitro transcription. HEK‐293T cells were transfected with unmodified mEGFP or m1Ψ‐modified m1ΨEGFP (m1ΨEGFP‐5%, m1ΨEGFP‐10%, m1ΨEGFP‐20%, m1ΨEGFP‐50%, m1ΨEGFP‐75%, and m1ΨEGFP‐100%). The expression and duration of EGFP were assessed using flow cytometry over a 6‐day period. As depicted in Figure 1A (upper part), m1ΨEGFP‐5%, m1ΨEGFP‐10%, and m1ΨEGFP‐20% group exhibited a higher percentage of EGFP‐positive cells (EGFP^+^cells %) and mean fluorescence intensity (MFI) compared to mEGFP group. Conversely, m1ΨEGFP‐50%, m1ΨEGFP‐75%, and m1ΨEGFP‐100% group had lower EGFP^+^cells % and MFI. Notably, the EGFP^+^cells % and MFI of cells transfected with m1ΨEGFP‐50%, m1ΨEGFP‐75%, and m1ΨEGFP‐100% were barely undetectable on day 6. EGFP expression detected by Western Blot on days 3, 4, and 5 was consistent with the flow cytometry (Figure 1A, bottom part, and Figure S1A). In summary, our finding indicates that the m1Ψ modification ratio might impact both mRNA translational ability and duration in HEK‐293T cells.

**FIGURE 1:**
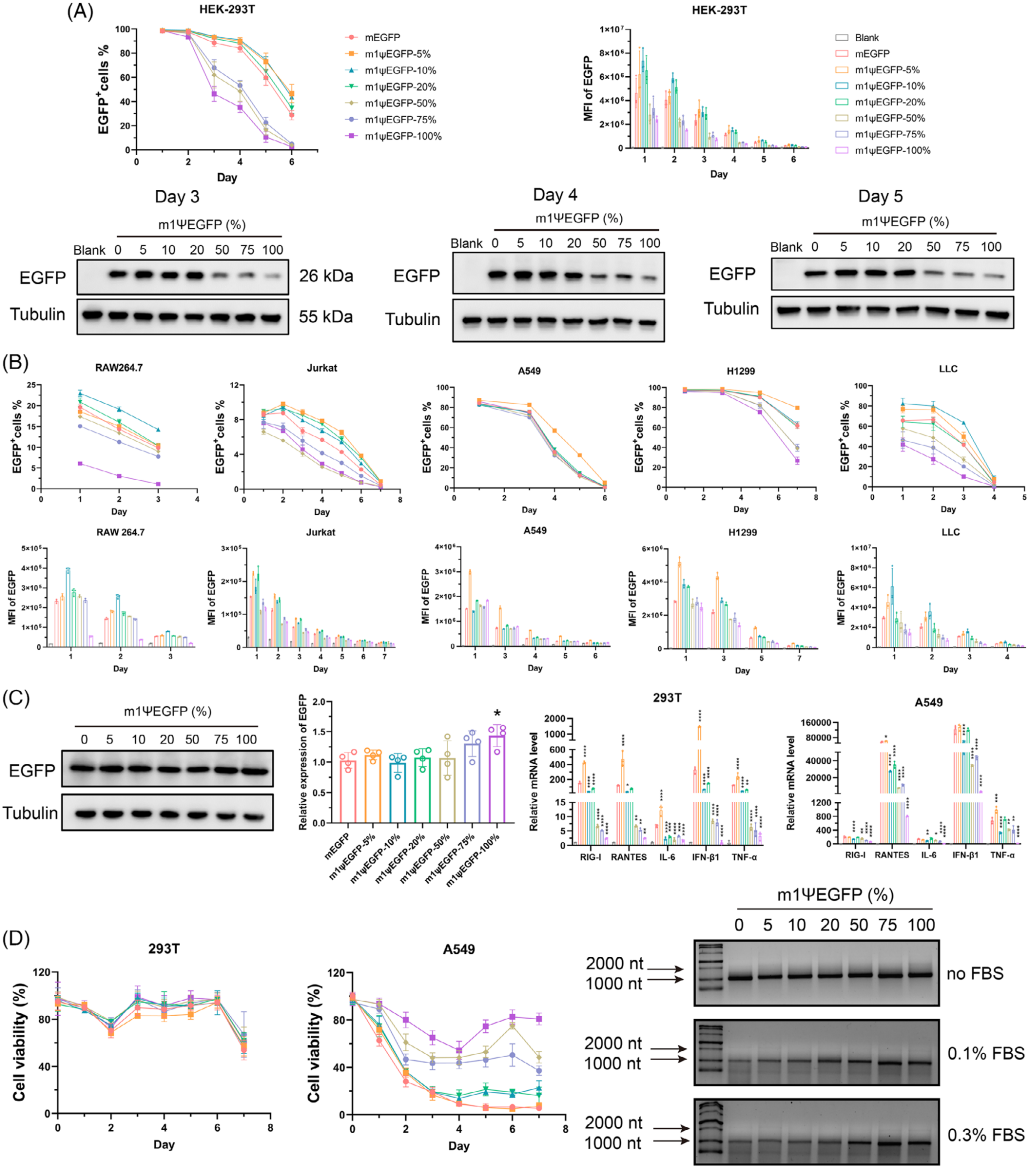
The influence of m1Ψ modification degree on messenger RNA (mRNA) protein expression, immunogenicity, and stability. (A, upper part) The rate of enhanced green fluorescent protein (EGFP)‐positive cells and mean fluorescence intensity measured by flow cytometry in HEK‐293T cells transfected with mRNA encoding EGFP (mEGFP) and different m1Ψ‐modified m1ΨEGFP. (A, bottom part) Western blot analysis of EGFP expression in HEK‐293T cells transfected with mEGFP and various m1Ψ‐modified m1ΨEGFP on day 3, day 4, and day 5. (B) The rate of EGFP‐positive cells and mean fluorescence intensity were measured by flow cytometry in RAW264.7 cells, Jurkat cells, A549 cells, H1299 cells, and LLC cells after transfected with mEGFP and different m1Ψ‐modified m1ΨEGFP. (C, left part) Cell‐free translation system for EGFP expression analysis. (C, right part) Evaluation of innate immunity activation by qPCR in HEK‐293T cells and A549 cells transfected with mEGFP and various m1Ψ‐modified m1ΨEGFP for 24 h. (D, left and middle part) Changes in cell viability of HEK‐293T cells and A549 cells after transfected with mEGFP and different m1Ψ‐modified m1ΨEGFP. (D, right part) Stability difference of mEGFP and different m1Ψ‐modified m1ΨEGFP after co‐incubated with fetal bovine serum. The lines of different colors in (B–D) represent the same group as (A). Data were presented as the mean ± SD. *p < 0.05, **p < 0.01, ***p < 0.001 and ****p < 0.0001, as determined by one‐way analysis of variance (ANOVA).

To validate the results in other cell types, we transfected RAW264.7 and Jurkat cells with unmodified and m1Ψ‐modified mRNA (Figure 1B, left part). In RAW264.7 cells, the m1ΨEGFP‐10% group exhibited the highest EGFP^+^cells % and MFI, whereas m1ΨEGFP‐100% showed the lowest. Similarly, in Jurkat cells, m1ΨEGFP‐5% exhibited the highest EGFP^+^cells % and MFI, while m1ΨEGFP‐50% had the lowest. Consistent with HEK‐293T cells, low m1Ψ ratio‐modified mRNA showed better protein expression and duration than unmodified, while high m1Ψ ratio resulted worse.

The impact of the m1Ψ modification ratio was further investigated in human A549, H1299 cells, and mouse LLC cells (Figure 1B, right part). In A549 cells, 5% m1Ψ modification showed the highest EGFP^+^cells % and MFI, while other ratios showed similar as unmodified. Not quite the same, in H1299 cells, 50%, 75%, and 100% m1Ψ modification decreased the EGFP^+^cells % and MFI compared to mEGFP. In LLC cells, m1ΨEGFP‐5% and m1ΨEGFP‐10% exhibited higher EGFP expression than mEGFP, while m1ΨEGFP‐50%, m1ΨEGFP‐75%, and m1ΨEGFP‐100% exhibited lower EGFP expression, with m1ΨEGFP‐100% had the lowest. To minimize the influence of cellular factors such as endocytosis, intracellular escape, transport, and degradation, we employed a cell‐free translation system to directly analyze protein translation. Interestingly, 75% and 100% modification tended to enhance protein translation, although only 100% modification had a significant difference (Figure 1C, left part). Notably, m1ΨEGFP‐5%, m1ΨEGFP‐10%, m1ΨEGFP‐20%, and m1ΨEGFP‐50% that exhibited enhanced protein expression in cells did not have higher protein yield in cell‐free system.

In the cell lines, we observed low m1Ψ modification ratio increased protein expression, whereas a high m1Ψ modification ratio decreased it. Although previous studies have demonstrated global m1Ψ modification enhanced protein expression,^3^ base modifications alter mRNA secondary structure, with translation alteration depending on the modified position. When m1Ψ global modifications occur in the 5′UTR region, formed stable structures inhibit translation, while increased protein expression occurs in CDS and 3′UTR region.^4^ Our results may be attributed to the preferential modified position of m1Ψ modification, resulting in a selective bias in modification position with different modification levels. Another explanation is that the slower decoding of m1Ψ‐modified sequences causes ribosome pause and collision, reducing polypeptide elongation rate and increasing frameshifted products.^5^ While m1ΨRNA targeting to endoplasmic reticulum membrane can relieve this, if without attachment, elongation arrest directly reduces translation efficiency. These suggest that post‐modified translation in eukaryotic systems is regulated by complex mechanisms. Nonetheless, under identical mRNA context, our results underscore the variation in protein expression across different m1Ψ modification levels, critical for optimizing modification ratios for diverse applications.

The mRNA immunogenicity influences its translation and stability. RIG‐I and TLR play crucial roles in activating innate immunity by recognizing exogenous RNA. To assess the immunogenicity, we transfected 293T cells and A549 cells with different modified mRNA and detected RIG‐I and TLR signaling activation by quantitative polymerase chain reaction (qPCR) (Figure 1C, right part). In 293T cells, a high m1Ψ modification ratio significantly reduced the mRNA levels of RIG‐I, RANTES, interleukin (IL)‐6, interferon (IFN)‐β1, and tumor necrosis factor‐α, with m1ΨEGFP‐100% having the lowest. Compared with mEGFP, m1ΨEGFP‐10%, and m1ΨEGFP‐20% also showed a reduction, while m1ΨEGFP‐5% markedly elevated. Similar trends were observed in A549 cells, while m1ΨEGFP‐5% did not elevate RIG‐I, IL‐6, and IFN‐β1. Collectively, these findings suggest that m1Ψ modification effectively reduces immunogenicity, thereby mitigating innate immune activation, particularly with a high modification ratio. However, it should be further explored why a high m1Ψ modification ratio reduces immunogenicity but does not enhance protein expression.

Subsequently, we examined the effect of m1Ψ modification on cell viability using CCK8 assay (Figure 1D, left part). In 293T cells, the lowest cell viability was observed in the 5% modification group, while no significant difference was observed among the other groups. In A549 cells, unmodified mEGFP significantly reduced the viability, which was effectively improved by m1Ψ modification (Figure 1D, middle part). Cell viability was positively correlated with modification ratio, consistent with altered immunogenicity. Considering that mRNA stability also affects translation in cells, we co‐incubated EGFR mRNA with different concentrations of fetal bovine serum to observe the impact of m1Ψ modification on stability. As displayed in Figure 1D (right part), m1Ψ modification protected mRNA from degradation, thereby increasing stability, and a high m1Ψ modification ratio had better stability. A similar result was observed in intracellular stability assay in mRNA‐transfected 293T cells by qPCR (Figure S1B).

In summary, our findings indicate that m1Ψ modification effectively reduces mRNA immunogenicity and enhances its stability, with a positive correction observed between modification ratio and stability. Compared to high‐ratio m1Ψ modification, such as 50%, 75% and 100%, low‐ratio m1Ψ modification exhibited higher protein translation efficiency. Furthermore, the relationship between protein expression level/duration and immunogenicity/stability is not linear. The limitation of this study is that only one mRNA sequence was used. Nevertheless, this study has significant implications for optimizing mRNA‐based therapies through modification strategies.

## AUTHOR CONTRIBUTIONS

Shaoyi Chen1 and Mantang Qiu designed the study and wrote the manuscript. Shaoyi Chen carried out the experiments and acquired the data. Shaoyi Chen, Zheng Liu, Jingsheng Cai, and Haoran Li analyzed the data and drew the figures. Mantang Qiu reviewed the manuscript and supervised the study. All authors have read and approved the manuscript.

## CONFLICT OF INTEREST STATEMENT

The authors declare no conflict of interest.

## ETHICS STATEMENT

Not applicable.

## Supporting information

Supporting Information

## Data Availability

The data that support the findings of this study are available from the corresponding author upon reasonable request.
