# Supplementary material for: N1‐methylpseudouridine modification level correlates with protein expression, immunogenicity, and stability of mRNA
Source: MedComm (2020). 2024 Sep 17;5(10):e691. doi: 10.1002/mco2.691 (PMC11406044; doi:10.1002/mco2.691)
Supplement: Supplementary file 1 — Supporting Information [file MCO2-5-e691-s001.docx]

Supplementary materials

**N1-methylpseudouridine modification level correlates with protein expression, immunogenicity, and stability of mRNA**

Shaoyi Chen^1, 2^, Zheng Liu^1, 2^, Jingsheng Cai^1, 2^, Haoran Li^1, 2^, Mantang Qiu^1, 2,^ *

1 Thoracic Oncology Institute & Research Unit of Intelligence Diagnosis and Treatment in Early Non-small Cell Lung Cancer, Department of Thoracic Surgery, Peking University People’s Hospital, Beijing 100044, China

2 Institute of Advanced Clinical Medicine, Peking University, Beijing 100191, China

**Materials and methods**

**mRNA synthesis and modification**

mRNA was synthesized through *in vitro* transcription (IVT), encompassing a 5' cap structure, a 5' untranslated region (UTR), a coding sequence (CDS), a 3' untranslated region, and a polyadenylate tail from 5' to 3' end. Initially, T7 promoter-5’UTR-EGFP-3’UTR sequences were amplified via PCR following digestion of the pUC57 plasmid. The PCR products were subsequently purified and recovered using a DNA purification kit (TIANGEN, DP214). For IVT, the purified products were added to transcription system (NEB, E2040S), consisting of T7 RNA polymerase mix, 10x T7 reaction buffer, ATP, UTP, CTP and GTP, and incubated in 37℃ for 2 h. Following treatment with Dnase I (NEB, M0303S) and RNA purification (NEB, T2040L), mRNA encoding EGFP (mEGFP) was obtained. Subsequently, a cap 1 structure was added to the 5’ end using vaccinia capping system (NEB, M2080S) and 2'-O-Methyltransferase (NEB, M0366S), then a polyadenylate tail was added to the 3’ end using E. coli Poly(A) Polymerase (NEB, M0276L). After these steps, the completed mEGFP was obtained. For m1Ψ modification, UTP was partially or completely replaced by m1Ψ during IVT, resulting in the generation of m1Ψ-modified mRNA variants, namely m1ΨEGFP-5%, m1ΨEGFP-10%, m1ΨEGFP-20%, m1ΨEGFP-50%, m1ΨEGFP-75% and m1ΨEGFP-100%.

**Cell transfection**

293T cells, RAW264.7 cells, Jurkat cells, A549 cells, H1299 cells, or LLC cells were seeded in 12-well or 6-well plates and cultured for 24 h. Subsequently, cells were transfected with 0.5 μg of mEGFP, m1ΨEGFP-5%, m1ΨEGFP-10%, m1ΨEGFP-20%, m1ΨEGFP-50%, m1ΨEGFP-75% or m1ΨEGFP-100%. Transfection was performed using Lipofectamine RNAiMAX reagent (Invitrogen, 13778150) following the manufacturer's instruction. In brief, RNAiMAX reagent and RNA were separately diluted in Opti-MEM medium and then mixed at a 1:1 (v/v) ratio. After a 5-minute incubation period, the RNA-lipid mixture was added to the cells. Cells were collected for analysis after incubating for the required experimental duration.

**Flow cytometry analysis**

Cells in the culture plate were detached and collected at various time points by centrifugation (1000 rpm, 5 min). After washing 3 times with PBS, cells were fixed in paraformaldehyde for 15 min. Subsequently, flow cytometry was employed to analyze EGFP+ cells % and MFI of EGFP using the FITC channel.

**Western blotting analysis**

To assess EGFP expression, cells in the culture plates were washed 3 times with chilled PBS and then lysed on ice for 30 min. Subsequently, cell lysates were centrifuged at 12000 rpm for 30 min to remove cellular debris. Protein supernatants were quantified and subjected to analysis by western blotting. In brief, proteins were separated by electrophoresis on an SDS-PAGE gel after being loaded at equal quantities, and then transferred to a PVDF membrane. The PVDF membrane was then sealed with 5% skim milk for 1 h, followed by overnight incubation with an anti-GFP primary antibody (Proteintech, 50430-2-AP). After washing with TBST, PVDF membrane was incubated with secondary antibody for 2h at room temperature. Finally, protein bands were visualized using chemiluminescence, and protein expression levels were semi-quantified by grayscale analysis.

**Cell-free translation system**

Cell-free translation system is utilized for the protein expression of mRNA transcribed *in vitro* or isolated from tissues or cells. Rabbit reticulocyte lysate (Promega, L4960) provides the necessary components for *in vitro* translation along with amino acids. Overall, 0.5 μg of mEGFP or different m1ΨEGFP variants was mixed with rabbit reticulocyte lysate, amino acid mixture, and RNasin ribonuclease inhibitor (Promega, N2511) respectively. The mixture was then subjected to a translation reaction incubation at 37°C for 90 min. Following the completion of translation reaction, a portion of the reaction solution was extracted for western blotting analysis.

**Quantitative real-time PCR (qPCR)**

293T cells and A549 cells were cultured in 6-well plates and transfected with mEGFP and different m1ΨEGFP variants. 24 h later, total RNA was extracted using Trizol reagent, followed by reverse transcription to cDNA using PrimeScript RT Master Mix (Takara, RR036A). For qPCR, the reaction mixture comprised 2x SYBR Mix (Vazyme, Q321), primers (10 μM), and the cDNA template. The PCR procedure involve a 30-s pre-degeneration step at 95°C, followed by 40 cycles of degeneration at 95℃ for 10 s and annealing/extension at 60°C for 30 s. The data were analyzed by method of 2^−ΔΔCT^ using GAPDH as the reference gene.

**CCK8 assay**

293T cells and A549 cells were seeded in 96-well plates and allowed to adhere for 24 h. Subsequently, they were transfected with either mEGFP or different m1ΨEGFP variants. At different time points post-transfection, 10 μl of CCK8 reagent was added to each well in the plates, followed by incubation for 1 h. Afterwards, the absorbance was measured at 450 nm using a microplate reader to calculate cell viability.

**mRNA stability assay**

FBS was utilized to assess the mRNA stability owing to its inherent RNase activity. Initially, 0.4 μg of mEGFP, m1ΨEGFP-5%, m1ΨEGFP-10%, m1ΨEGFP-20%, m1ΨEGFP-50%, m1ΨEGFP-75% or m1ΨEGFP-100% was co-incubated with either ddH_2_O, 0.1% FBS, or 0.3% FBS for 10 min at 37℃. Subsequently, the reaction solutions were analyzed by agarose gel electrophoresis.

To assess the intracellular stability, 293T cells were cultured in 6-well plates and transfected with mEGFP and different m1ΨEGFP variants. After 6 h internalization, total RNA was extracted at various time intervals (day 0, day 1, and day 3). The relative levels of EGFP mRNA were detected using qPCR and normalized to day 0. GAPDH was used as the reference gene.

**Supplementary Figure and Figure Legend**


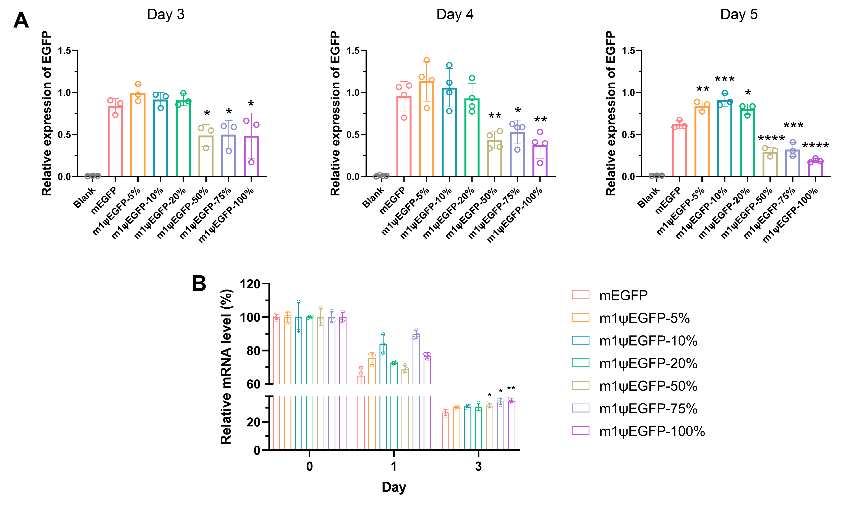


**Figure S1. (A)** WB statistical analysis of EGFP expression in 293T cells transfected with mEGFP and different m1ΨEGFP variants on day 3, day 4, day 5. **(B)** The intracellular stability of various mRNA measured by qPCR, data were presented as mean ± SD (n=3). **p* < 0.05, ***p* < 0.01, ****p* < 0.001 and *****p* < 0.0001, compared to mEGFP group.

**Sequence of mEGFP**

taatacgactcactataggggggaaataagagagaaaagaagagtaagaagaaatataagagccaccgccaccatggtgagcaagggcgaggagctgttcaccggggtggtgcccatcctggtcgagctggacggcgacgtaaacggccacaagttcagcgtgtctggcgagggcgagggcgatgccacctacggcaagctgaccctgaagttcatctgcaccaccggcaagctgcccgtgccctggcccaccctcgtgaccaccctgacctacggcgtgcagtgcttcagccgctaccccgaccacatgaagcagcacgacttcttcaagtccgccatgcccgaaggctacgtccaggagcgcaccatcttcttcaaggacgacggcaactacaagacccgcgccgaggtgaagttcgagggcgacaccctggtgaaccgcatcgagctgaagggcatcgacttcaaggaggacggcaacatcctggggcacaagctggagtacaactacaacagccacaacgtctatatcatggccgacaagcagaagaacggcatcaaggcgaacttcaagatccgccacaacatcgaggacggcagcgtgcagctcgccgaccactaccagcagaacacccccatcggcgacggccccgtgctgctgcccgacaaccactacctgagcacccagtccgccctgagcaaagaccccaacgagaagcgcgatcacatggtcctgctggagttcgtgaccgccgccgggatcactctcggcatggacgagctgtacaagtaagctggagcctcggtggccatgcttcttgccccttgggcctccccccagcccctcctccccttcctgcacccgtacccccgtggtctttgaataaagtctgagtgggcggca

*Red: T7 promoter; Green: 5’UTR; Orange: Kozak sequence; Blue: CDS of EGFP; Purple: 3’UTR.*

**Supplementary table 1. Sequence of primers in qPCR**

| **Gene** | **Primer-F** | **Primer-R** |
| --- | --- | --- |
| RIG-I | GGAAGACCCTGGACCCTACCTA | AAAAGCCACGGAACCAGCC |
| RANTES | CTTGATGTGGGCACGGGGCAGTG | GGCAGCCCTCGCTGTCATCCTCA |
| IL-6 | TGTGAAAGCAGCAAAGAGGC | CAAACTCCAAAAGACCAGTGATG |
| IFN-β1 | CCAACAAGTGTCTCCTCCAAA | TCCTGTCCTTGAGGCAGTATTC |
| TNF-α | CGAGTGACAAGCCTGTAGCC | TGAAGAGGACCTGGGAGTAGAT |
| mEGFP | GGATCACTCTCGGCATGGAC | TGCCGCCCACTCAGACTTTA |
| GAPDH | CGCTCTCTGCTCCTCCTGTTC | ATCCGTTGACTCCGACCTTCAC |
